# Use of electroconvulsive therapy in Nordic and western European countries

**DOI:** 10.1192/j.eurpsy.2026.12238

**Published:** 2026-07-29

**Authors:** Richard Braithwaite, Ali Amad, Carlo Ignazio Cattaneo, Jan Di Pauli, Martha Finnegan, Pedro Frias, Manuel Gonçalves-Pinho, Michael Grözinger, Kaija Järventausta, Anders Jørgensen, Saana Karttunen, Ute Kessler, Pia Nordanskog, Jasmien Obbels, Albino J. Oliveira-Maia, Didi Rhebergen, Pascal Sienaert, Floris Quirijn Tas, Saskia Wilhelmy, George Kirov

**Affiliations:** 1 Department of Clinical Neuroscience, Brighton and Sussex Medical School, University of Sussex, Brighton, UK; 2 Meadowfield Hospital, Sussex Partnership NHS Foundation Trust, Worthing, UK; 3 Inserm, CHU Lille, U1172, LilNCog, Lille Neuroscience & Cognition, University of Lille, Lille, France; 4 Department of Neuroimaging, Institute of Psychiatry, Psychology and Neuroscience, King’s College London, London, UK; 5 Department of Mental Health, ASL Biella, Biella, Italy; 6 Department of Adult Psychiatry, Rankweil Regional Hospital, Rankweil, Austria; 7 Department of Psychiatry, Trinity College Dublin, Dublin, Ireland; 8 Department of Psychiatry, Tallaght University Hospital, Dublin, Ireland; 9 Departamento de Saúde Mental, Unidade Local de Saúde do Baixo Alentejo, Beja, Portugal; 10 Escola Nacional de Saúde Pública, Universidade Nova de Lisboa, Lisbon, Portugal; 11 Department of Clinical Neurosciences and Mental Health, Faculty of Medicine, University of Porto, Porto, Portugal; 12 Department of Psychiatry and Mental Health, Unidade Local de Saúde Tâmega e Sousa, Penafiel, Portugal; 13 Department of Psychiatry, Psychotherapy and Psychosomatics, Faculty of Medicine, RWTH Aachen University, Aachen, Germany; 14 Faculty of Medicine and Health Technology, Tampere University, Tampere, Finland; 15 Department of Psychiatry, Tampere University Hospital, Tampere, Finland; 16 Department of Clinical Medicine, University of Copenhagen, Copenhagen, Denmark; 17 Psychiatric Centre Copenhagen, Frederiksberg, Denmark; 18 Department of Psychiatry, Oulu University Hospital, Wellbeing Services County of Northern Ostrobothnia, Finland; 19 Research Unit of Population Health, University of Oulu, Oulu, Finland; 20 Department of Clinical Medicine, University of Bergen, Bergen, Norway; 21 Division of Psychiatry, Haukeland University Hospital, Bergen, Norway; 22 Centre for Social and Affective Neuroscience, Department of Clinical and Experimental Medicine, Faculty of Health Sciences, Linköping University, Linköping, Sweden; 23 Department of Psychiatry, Region Östergötland, Linköping, Sweden; 24 University Psychiatric Center KU Leuven, Academic Center for ECT and Neuromodulation (AcCENT), Kortenberg, Belgium; 25 Champalimaud Clinical Centre, Neuroscience of Disease Programme & Digital Neurotherapeutics Centre, Champalimaud Foundation, Lisbon, Portugal; 26 Nova Medical School (NMS), Faculdade de Ciências Médicas (FCM), Universidade Nova de Lisboa, Lisbon, Portugal; 27 Mental Health Care Institute, GGz Centraal, Amersfoort, Netherlands; 28 Department of Psychiatry, Amsterdam UMC, Vrije Universiteit, Amsterdam, Netherlands; 29 Department of Psychiatry, Donders Institute for Brain, Cognition and Behaviour, Nijmegen, Netherlands; 30 Department of Psychiatry, Radboud University Medical Center, Nijmegen, Netherlands; 31 Institute for the History of Medicine, Carl Gustav Carus Faculty of Medicine, Dresden University of Technology, Dresden, Germany; 32 Division of Psychological Medicine and Clinical Neuroscience, School of Medicine, Cardiff University, Cardiff, UK

**Keywords:** depression, electroconvulsive therapy, Europe, international comparison, Scandinavia

## Abstract

**Background:**

Electroconvulsive therapy (ECT) is a safe and effective medical treatment, yet provision varies internationally. We provide an up-to-date review of ECT practice in Nordic and western European countries.

**Methods:**

European experts contributed data from peer-reviewed literature, governmental publications, unpublished administrative datasets, and hitherto unpublished surveys. The primary outcome was the number of individuals treated with ECT in each country per year. Primary analysis compared the annual treated person rate per million population in the most recent year since 2015 for which data were available. Secondary analysis compared trends in rates over 25 years, in countries for which a reliable outcome was available at two or more time points.

**Results:**

Across 15 countries, 12 had data meeting primary analysis criteria. Of these, all Nordic countries had annual treated patient rates per million population over 200, Italy 2.7, and other western European countries between 30 and 100. German-, Dutch-, and French-speaking countries saw increases in ECT use over 25 years, while English-speaking countries exhibited declines. Unilateral placement varied from 16% of treatments in the United Kingdom to 79% in Norway. Everywhere, most ECT was given for depressive episodes.

**Conclusions:**

Although the optimal rate of use is unknown, widely differing rates across European nations suggest ECT is underused in some high-income countries, depriving patients of vital medical care. The European ECT community must support the development of reliable data collection on usage across the continent and service improvement where provision is underdeveloped, to ensure appropriate treatment is offered to all patients for whom it is indicated.

## Introduction

Electroconvulsive therapy (ECT) is a safe and well-tolerated medical treatment for several psychiatric and neurological disorders [1]. It is the most effective intervention in depressive episodes [2, 3] and is also variously used in the treatment of mania [4], schizophrenia [5], and catatonia [6].

However, provision varies markedly worldwide [7]. A 2012 review, based on published data from various locations, estimated annual treated person rates (ATPRs) for several nations [7]. While these included some Nordic and western European countries, only the calculations for Denmark, Germany, Norway, Spain, Sweden, and the United Kingdom were correct and based on national-level data, most of which are now two decades old. Use across Nordic countries was last documented three decades ago [8]. Recent reviews have covered the Baltic states [9] and central and eastern Europe [10], the latter showing ATPRs varying from 2 per million inhabitants in Ukraine to 292 in Slovakia.

The present study aims to provide an up-to-date review of ECT practice in the remainder of the continent. We present an overview of use across Nordic and western European countries, using recent national-level data to calculate each nation’s ATPR and combining this with more historical data, where available, to identify trends in usage over time.

## Method

### Inclusion criteria

An expert on each country was identified via informal networks and asked to collaborate if they had access to national data on ECT usage (unless data were from before 1 January 2015 or from selected regions comprising under half the country’s population), or could collect novel data for the present study. For some countries, it was appropriate to involve more than one individual, to complement skills, knowledge, and resources. National psychiatric associations were not directly contacted. Countries with a population under one million were excluded, to avoid effects of random fluctuations in low numbers.

### Outcomes

The primary outcome was the number of individuals treated with ECT in any given year. Further information was sourced, where available, on the number of ECT centres, attitudes and access to ECT, national policy guidance, diagnostic indications, and treatment parameters, such as electrode placement and hospital admission status.

### Data sources

Sources included peer-reviewed literature, governmental publications not subject to peer review, published and unpublished administrative datasets, and hitherto unpublished surveys, methodologies of which are detailed in Supplementary Material.

### Analysis

The primary analysis compared ATPRs (calculated by dividing the number of individuals treated by the national population in millions that year, sourced from https://data.worldbank.org) for the most recent year for which data were available in each country. For surveys reliant on optional responses from treatment providers, extrapolation of data to cover non-respondent providers was considered if the response rate exceeded 50%. Secondary analyses compared mean number of patients per centre and, in countries for which the primary outcome was available at two or more time points, trends in ATPRs over 25 years and compound annual growth rate (CAGR), calculated using this formula [11]:

$$\text{CAGR}={\left(\frac{{\text{ATPR}}_{\text{last}}}{{\text{ATPR}}_{\text{first}}}\right)}^{\frac{1}{\left({\text{year}}_{\text{last}}-{\text{year}}_{\text{first}}\right)}}-1$$

### Ethical approval

As this study was based on retrospective analysis of anonymised data and did not require treatment providers or other organizations to access any individual patient records, ethical approval was not required in any of the participating countries.

## Results

**Figure 1.**
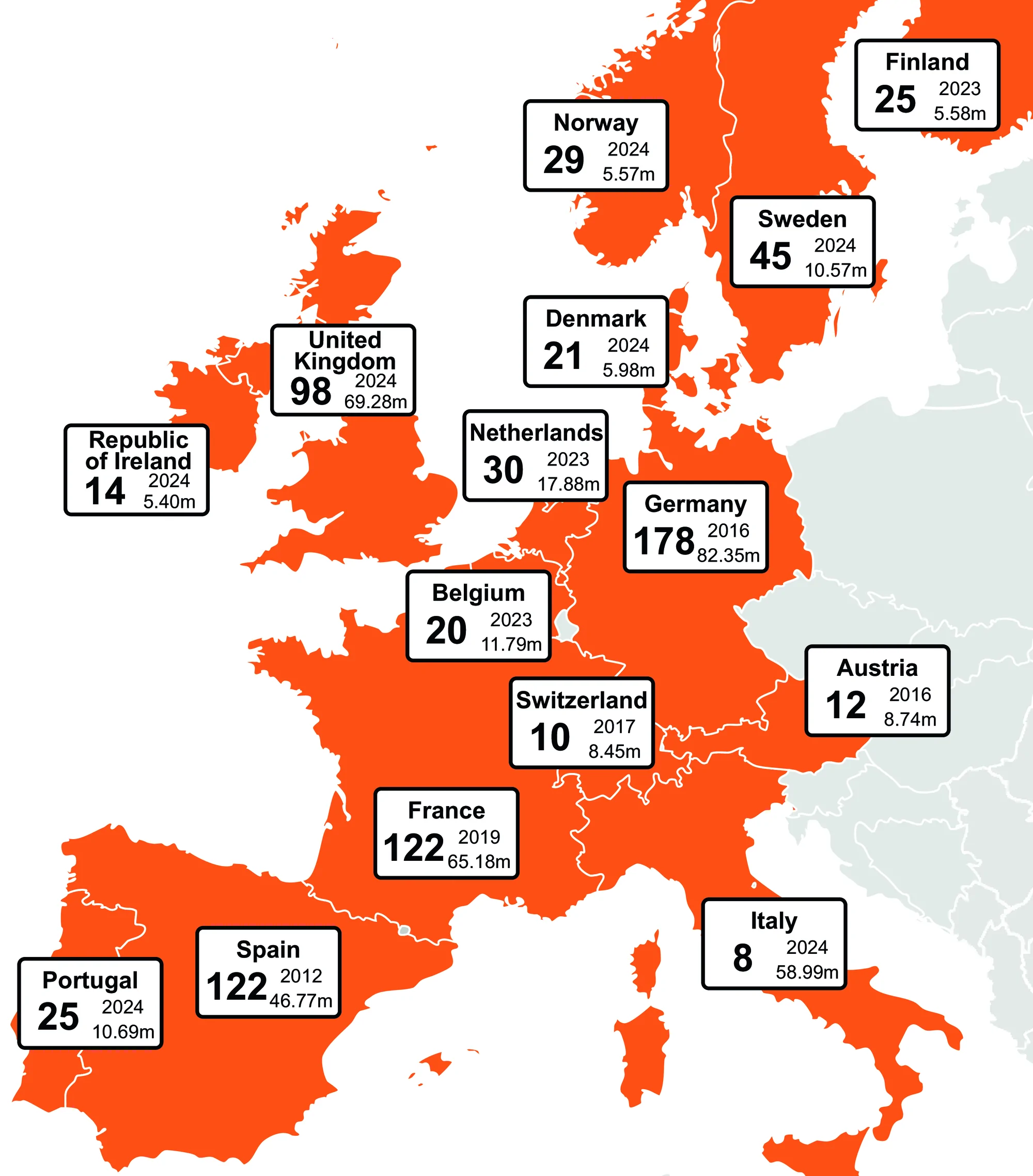
Number of electroconvulsive therapy centres. *Note*: Estimated number of centres in each country shown in bold font. Year of data and national population in that year shown in standard font. Figure 1. long description.

**Figure 2.**
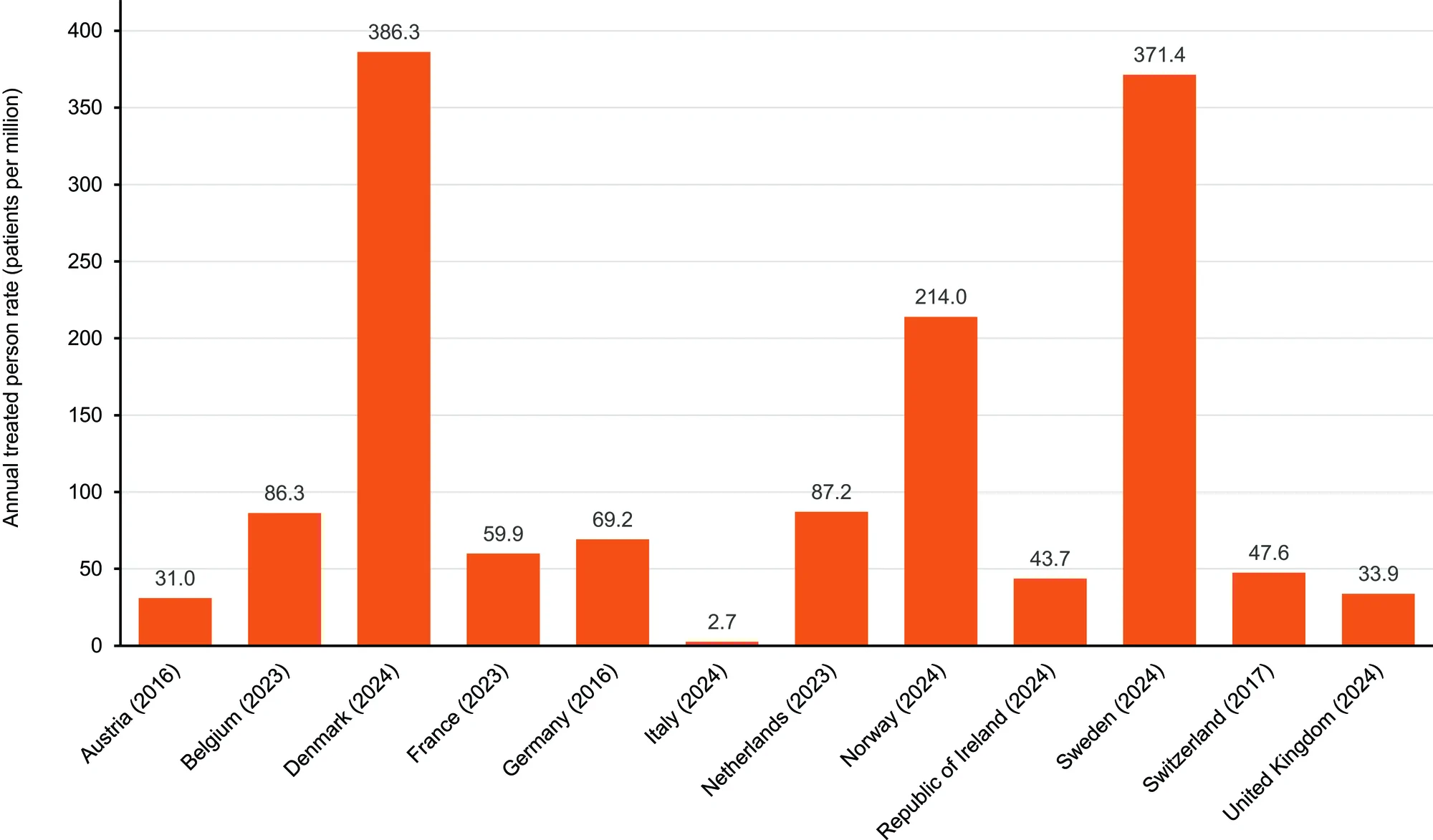
Annual treated person rate. *Note*: Based on the most recent year with available data. Exclusions: Finland (low response rate for most recent survey), Portugal (data only from public hospital inpatients), and Spain (most recent data from before 2015). Figure 2. long description.

**Figure 3.**
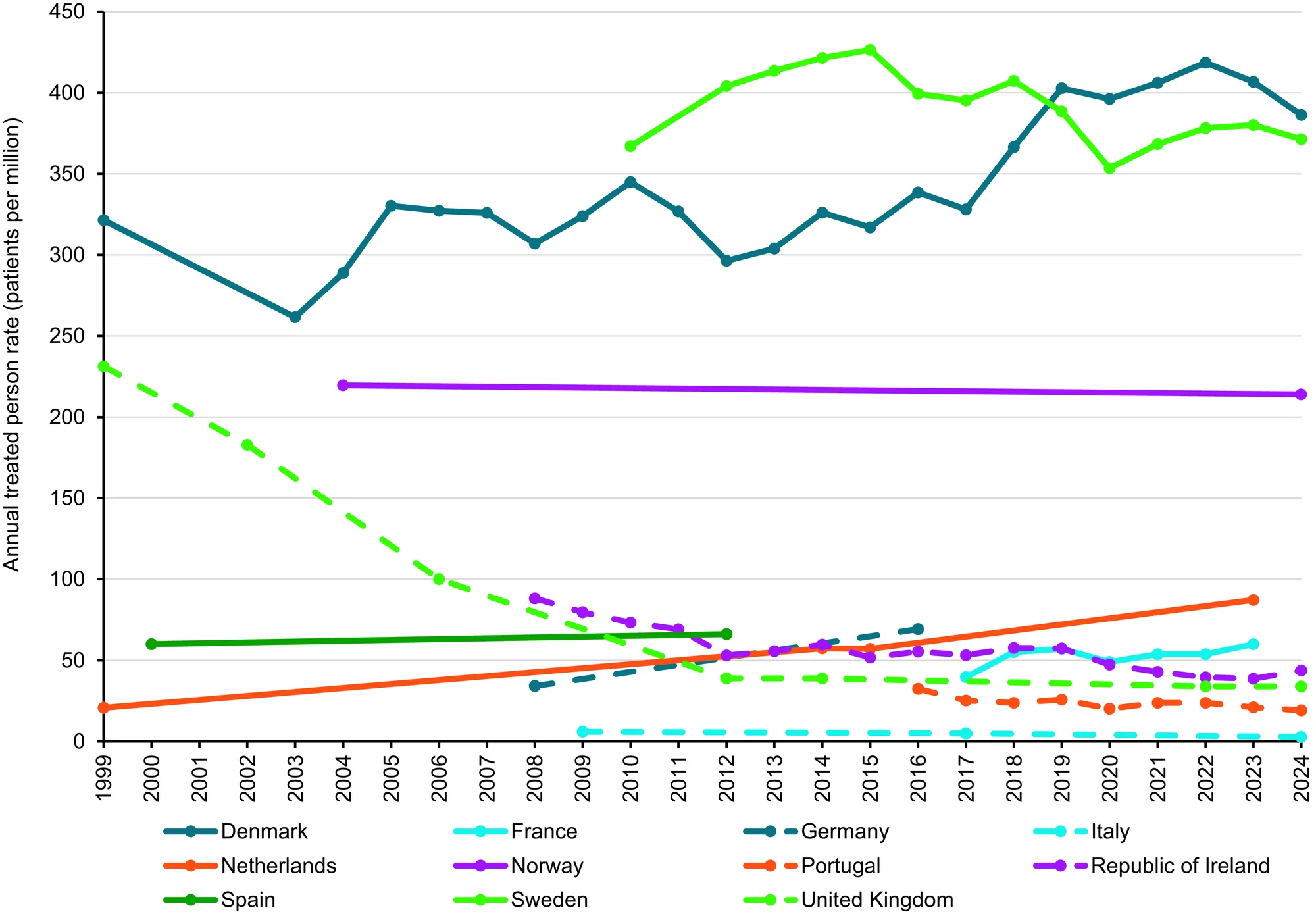
Trends in electroconvulsive therapy use over 25 years. *Note*: Exclusions: Austria and Belgium (data only available for one time point); Finland (low response rate for most recent survey). Figure 3. long description.

**Figure 4.**
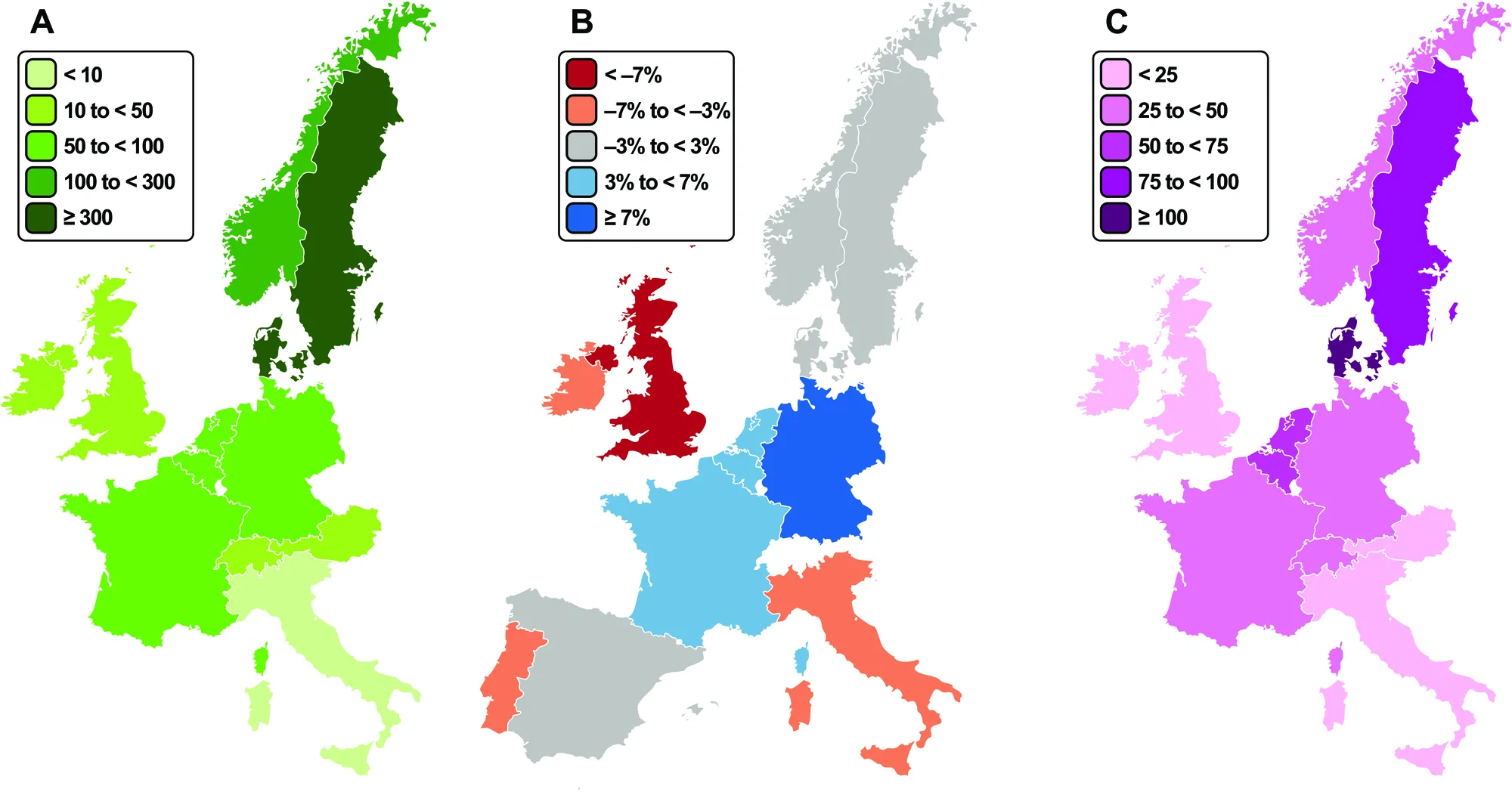
Use of ECT in Nordic and western European countries. *Note*: (A) ECT annual treated person rate per million population. Data from the most recent year with available data. Exclusions: Finland (low response rate for most recent survey), Portugal (data only from public hospital inpatients), and Spain (most recent data from before 2015). (B) Compound annual growth rate in ECT use. Values calculated using different time periods between 1999 and 2024, due to varying data availability. Belgium value based on numbers of treatments, not numbers of patients. Exclusions: Austria and Switzerland (data only available for one time point); Finland (low response rate for most recent survey). (C) Mean number of patients treated per ECT centre per year. Same exclusions as Panel A. ECT, electroconvulsive therapy. Figure 4. long description.

There are 15 Nordic or western European countries with populations over one million; numbers of ECT centres in each are shown in Figure 1. Usage data were available for at least 1 year since 2015 for 14 countries, excluding Spain. Finland (survey response rate <50%) and Portugal (data restricted to public hospital inpatients) were excluded from the primary analysis. Sources of recent and historical data are shown in Table 1. Results are detailed below by country and in Table 1, Figures 2–4, and Supplementary Material.

**Table 1.** Sources of data and estimated numbers of patients treated annually Table 1. long description.

Year of data	Reference	Data source	Coverage and details		Estimated patients per year	Population (m)	ATPR (patients per million)
**Austria**							
2016	Unpublished^a^	Survey	100% response from known ECT centres		271	8.74	31.0
**Belgium**							
2003	Sienaert et al. [12]	Insurance data	Full coverage; *n* (treatments) = 7,068		nc		
2013	Sienaert et al. [13]	Insurance data	Full coverage; *n* (treatments) = 11,475		nc		
2023	NIHDI, pc, 11 Dec 2024	Insurance data	Full coverage; *n* (treatments) = 15,046		nc		
2023	Unpublished^a^	Survey	6 largest ECT centres, accounting for 11,705 (77.8%) of 15,046 treatments in 2023 insurance dataset (above); *n* (patients) = 792, extrapolated using divisor of 0.778		1,018	11.79	86.3
**Denmark**							
1999	Andersson & Bolwig [14]	Survey	100% response		1,710	5.32	321.4
2003	DNPR, pc, 5 Mar 2026	National register	Full coverage		1,410^b^	5.39	261.6
2004	DNPR, pc, 5 Mar 2026	National register	Full coverage		1,560^b^	5.40	288.9
2005	DNPR, pc, 5 Mar 2026	National register	Full coverage		1,790^b^	5.42	330.3
2006	DNPR, pc, 5 Mar 2026	National register	Full coverage		1,780^b^	5.44	327.2
2007	DNPR, pc, 5 Mar 2026	National register	Full coverage		1,780^b^	5.46	326.0
2008	Hundrup et al. [15]	National register	Full coverage		1,685	5.49	306.9
2009	Hundrup et al. [15]	National register	Full coverage		1,788	5.52	323.9
2010	Hundrup et al. [15]	National register	Full coverage		1,914	5.55	344.9
2011	Hundrup et al. [15]	National register	Full coverage		1,820	5.57	326.8
2012	Hundrup et al. [15]	National register	Full coverage		1,657	5.59	296.4
2013	Hundrup et al. [15]	National register	Full coverage		1,705	5.61	303.9
2014	Hundrup et al. [15]	National register	Full coverage		1,839	5.64	326.1
2015	DNPR, pc, 5 Mar 2026	National register	Full coverage		1,800^b^	5.68	316.9
2016	DNPR, pc, 5 Mar 2026	National register	Full coverage		1,940^b^	5.73	338.6
2017	DNPR, pc, 5 Mar 2026	National register	Full coverage		1,890^b^	5.76	328.1
2018	DNPR, pc, 5 Mar 2026	National register	Full coverage		2,100^b^	5.73	366.5
2019	DNPR, pc, 5 Mar 2026	National register	Full coverage		2,340^b^	5.81	402.8
2020	DNPR, pc, 5 Mar 2026	National register	Full coverage		2,310^b^	5.83	396.2
2021	DNPR, pc, 5 Mar 2026	National register	Full coverage		2,380^b^	5.86	406.1
2022	DNPR, pc, 5 Mar 2026	National register	Full coverage		2,470^b^	5.90	418.6
2023	DNPR, pc, 5 Mar 2026	National register	Full coverage		2,420^b^	5.95	406.7
2024	DNPR, pc, 5 Mar 2026	National register	Full coverage		2,310^b^	5.98	386.3
**Finland**							
2013	Sumia et al. [16]	Survey	25 (86%) of 29 ECT centres, *n* (patients) = 1,023, extrapolated		1,260	5.44	231.6
2023	Unpublished^a^	Survey	8 (32%) of 25 ECT centres, *n* (patients) = 840 (26 in national forensic unit and 814 in 7 regional units) covering 2.21 m population [39.5% of national population], extrapolated using divisor of 0.395 to 2,061)		2,087	5.58	374.0^c^
**France**							
2017	Barruel et al. [17]	Health claims registry	100% coverage		2,562	64.74^d^	39.6
2018	Barruel et al. [17]	Health claims registry	100% coverage		3,573	64.97^d^	55.0
2019	Barruel et al. [17]	Health claims registry	100% coverage		3,720	65.18^d^	57.1
2020	Barruel et al. [17]	Health claims registry	100% coverage		3,200	65.39^d^	48.9
2021	Barruel et al. [17]	Health claims registry	100% coverage		3,530	65.68^d^	53.7
2022	Barruel et al. [17]	Health claims registry	100% coverage		3,547	66.01^d^	53.7
2023	Barruel et al. [17]	Health claims registry	100% coverage		3,972	66.27^d^	59.9
**Germany**							
2008	Loh et al. [18]	Survey	107 (58%) of 183 ECT centres; *n* (patients) = 1,863, extrapolated by comparing consumption of disposable equipment in responding and nonresponding centres		2,800	82.11	34.1
2016	Jolivet & Grözinger [19]	Survey	130 (73.0%) of 178 ECT centres, accounting for 79.6% of psychiatric beds; *n* (patients) = 4,330, extrapolated using a divisor of the midpoint between 0.730 and 0.796		5,700	82.35	69.2
2023	Dreger et al. [20]	Reimbursement data	Full coverage; *n* (treatments) = 72,193		nc		
**Italy**							
2009	Abbiati et al. [21]	Telephone survey	100% response; composite of data from different years between 1999 and 2009 for each ECT centre		350	59.56	5.9
2017	Wilhelmy et al. [22]	Survey	100% response		293	60.00	4.9
2024	Unpublished^a^	Survey	7 (88%) of 8 ECT centre; *n* (patients) = 99; missing centre in 2024 accounted for 37.54% of all patients in 2017 (Wilhelmy et al., 2020), so extrapolated using a divisor of 0.6246		159	58.99	2.7
**Netherlands**							
1999	Verwey & Sienaert [23]	Mandatory national register	Full coverage; data from Chief Medical Inspectorate for Mental Health		328	15.81	20.7
2008	van Waarde et al. [24]	Survey	94% response; *n* (treatments) = 13,500		nc		
2014	van den Dungen [25]	Health insurance data	Full coverage		966	16.87	57.3
2015	van den Dungen [25]	Health insurance data	Full coverage		966	16.94	57.0
2015	Verwey et al. [26]	Survey	100% response; *n* (treatments) = 15,633		nc		
2023	Vektis, pc, 5 Nov 2024	Health insurance data	Full coverage		1,559	17.88	87.2
**Norway**							
2004	Schweder et al. [27]	Survey	67% response; data extrapolated		1,008	4.59	219.6
2024	NPR, pc, 10 Dec 2025; Unpublished^a^	National register; plus survey	28 (97%) of 29 ECT centres responded to survey		1,192	5.57	214.0
**Portugal**							
2016	INE [28]	National IP register	Full coverage of public hospitals		335	10.33	32.4^e^
2017	INE [28]	National IP register	Full coverage of public hospitals		260	10.30	25.2^e^
2018	INE [28]	National IP register	Full coverage of public hospitals		244	10.28	23.7^e^
2019	INE [28]	National IP register	Full coverage of public hospitals		265	10.29	25.8^e^
2020	INE [28]	National IP register	Full coverage of public hospitals		207	10.30	20.1^e^
2021	INE [28]	National IP register	Full coverage of public hospitals		247	10.36	23.8^e^
2022	INE [28]	National IP register	Full coverage of public hospitals		247	10.43	23.7^e^
2023	INE [28]	National IP register	Full coverage of public hospitals		222	10.58	21.0^e^
2024	INE [28]	National IP register	Full coverage of public hospitals		204	10.69	19.1^e^
**Republic of Ireland**							
2008	MHC [29]	Mandatory register	100% coverage		396	4.49	88.2
2009	MHC [29]	Mandatory register	100% coverage		362	4.54	79.7
2010	MHC [29]	Mandatory register	100% coverage		334	4.56	73.2
2011	MHC [29]	Mandatory register	100% coverage		316	4.58	69.0
2012	MHC [29]	Mandatory register	100% coverage		244	4.60	53.0
2013	MHC [29]	Mandatory register	100% coverage		257	4.62	55.6
2014	MHC [29]	Mandatory register	100% coverage		278	4.66	59.7
2015	MHC [29]	Mandatory register	100% coverage		243	4.70	51.7
2016	MHC [29]	Mandatory register	100% coverage		263	4.76	55.3
2017	MHC [29]	Mandatory register	100% coverage		257	4.83	53.2
2018	MHC [29]	Mandatory register	100% coverage		282	4.90	57.6
2019	MHC [29]	Mandatory register	100% coverage		286	4.98	57.4
2020	MHC [29]	Mandatory register	100% coverage		239	5.04	47.4
2021	MHC [29]	Mandatory register	100% coverage		219	5.11	42.9
2022	MHC [29]	Mandatory register	100% coverage		206	5.21	39.5
2023	MHC [29]	Mandatory register	100% coverage		205	5.31	38.6
2024	MHC [29]	Mandatory register	100% coverage		236	5.40	43.7
**Spain**							
2000	Bertolín-Guillén et al. [30]	Survey	100% response		2,435	40.57	60.0^f^
2012	Vera et al. [31]	Survey	207 (93%) of 222 hospitals responded, of which 122 used ECT; true number of ECT centres not known, precluding extrapolation		3,090	46.77	66.1^f^
**Sweden**							
2010	Cited in Nordanskog et al. [32]	Government estimate	Unclear (not publicly available)		Not stated	9.38	367.0
2012	Q-ECT [33]	National register	100% coverage		3,848	9.52	404.2
2013	Q-ECT [33]	National register	100% coverage		3,972	9.60	413.5
2014	Q-ECT [33]	National register	100% coverage		4,087	9.70	421.5
2015	Q-ECT [33]	National register	100% coverage		4,179	9.80	426.5
2016	Q-ECT [33]	National register	100% coverage		3,963	9.92	399.4
2017	Q-ECT [33]	National register	100% coverage		3,976	10.06	395.3
2018	Q-ECT [33]	National register	100% coverage		4,144	10.18	407.3
2019	Q-ECT [33]	National register	100% coverage		3,993	10.28	388.5
2020	Q-ECT [33]	National register	100% coverage		3,660	10.35	353.5
2021	Q-ECT [33]	National register	100% coverage		3,836	10.42	368.5
2022	Q-ECT [33]	National register	100% coverage		3,966	10.49	378.2
2023	Q-ECT [33]	National register	100% coverage		4,005	10.54	380.1
2024	Q-ECT [33]	National register	100% coverage		3,926	10.57	371.4
**Switzerland**							
2017	Wilhelmy et al. [34]	Survey	38 (75%) of 51 hospitals responded, of which 10 used ECT		402	8.45	47.6
**United Kingdom**							
1999	Cited in Buley et al. [35]	Government survey	Unclear (no longer publicly available); England only		11,340	49.03^g^	231.3
2002	Cited in Buley et al. [35]	Government survey	unclear (no longer publicly available); England only		9,088	49.68^g^	182.9
2006	Bickerton et al. [36]	Survey	74% response; data extrapolated; methodology unclear; England only		5,104	50.97^g^	100.1
2012	Buley et al. [35]	Survey	74 (76%) of 98 ECT centres; *n* (patients) = 1,569, extrapolated using divisor of 0.755; England only		2,078	53.49^g^	38.8
2014	Buley et al. [35]	Survey	71 (80%) of 89 ECT centres; *n* (patients) = 1,680, extrapolated using divisor of 0.798; England only		2,106	54.32^g^	38.8
2022	SEAN [37]	Mandatory register	100% coverage; Scotland only		271	5.45^g^	49.7
2022	ECTAS, pc, 5 Mar 2026	Mandatory register	80 (99%) of 81 ECT centres; *n* (patients) = 1,999; extrapolated using divisor of 0.987; E/W/NI		2,024	62.15^g^	32.6
2022	SEAN [37]; ECTAS, pc, 5 Mar 2026	Mandatory registers	Combination of above Scotland and E/W/NI data (whole UK)		2,295	67.60^g^	33.9
2024	SEAN [38]	Mandatory register	100% coverage; Scotland only		264	5.55^g^	47.6
2024	ECTAS, pc, 5 Mar 2026	Mandatory register	82 (100%) of 82 ECT centres; *n* (patients) = 2,083; E/W/NI		2,083	63.74^g^	32.7
2024	SEAN [38]; ECTAS, pc, 5 Mar 2026	Mandatory registers	Combination of above Scotland and E/W/NI data (whole UK)		2,347	69.28^g^	33.9

Abbreviations: ECT, electroconvulsive therapy; ATPR, annual treated person rate; nc, not calculated; pc, personal communication; NIHDI, National Institute for Health and Disability Insurance; DNPR, Danish National Patient Register; NPR, Norwegian Patient Registry; INE, Instituto Nacional de Estatistíca; IP, inpatient; MHC, Mental Health Commission; Q-ECT, Kvalitetsregister ECT; SEAN, Scottish Electroconvulsive Therapy Audit Network; ECTAS, Electroconvulsive Therapy Accreditation Service; E/W/NI, England, Wales, and Northern Ireland.

a See Supplementary Materials for further information on methodology of previously unpublished surveys.

b Value rounded to the nearest 10 at the request of Statistics Denmark.

c Excluded from all comparative analyses due to low survey response rate.

d Population data for Metropolitan France from https://www.insee.fr/en/statistiques/serie/000067671 [accessed 4 March 2026].

e Excluded from primary comparative analysis due to restriction of scope to public hospital inpatients.

f Excluded from primary comparative analysis due to age of data.

g Regional and aggregate population data from https://www.ons.gov.uk/ [accessed 15 February 2026].

### Austria

A hitherto unpublished survey (Supplementary Material) identified 12, predominantly public, facilities in 2017, all of which responded, detailing 271 individuals receiving 2,682 treatments. Depressive episodes accounted for 86% of activity. Outpatient treatment was provided at two centres, and maintenance ECT at 10. Around 22% of treatments were given without consent, in accordance with legal provisions. Recent decades have seen expanding provision, with 7 ECT centres in 2000 [39], 8 in 2004 [40], and 10 in 2013 [41], while there were still 12 centres in 2026 (Baldinger-Melich P & Di Pauli J, personal communication, 9 March 2026).

### Belgium

For the present study, a survey of the six largest ECT centres revealed 11,705 treatments administered to 792 individuals in 2023 (Supplementary Material). Insurance data show these centres accounted for 77.8% of 15,046 ECT treatments nationally; hence, an estimated 1,018 individuals received ECT in 2023, 34% as outpatients. These data show the number of ECT facilities has reduced, from 32 in 2003 [12] to 20 in 2023, while the annual number of ECT treatments has more than doubled from 7,068 [13]. A nationwide survey [13] suggested ECT practice largely followed contemporary US [42] and Netherlands [43] guidelines. Striking regional differences exist: despite accounting for 42% of the population, the Wallonia and Brussels regions are collectively responsible for only 13% of ECT activity. In contrast, Flanders, with 58% of the population, accounts for 87%.

### Denmark

Individual treatments are recorded in the National Patient Registry [44] with 90% completeness, allowing for epidemiological studies into ECT use, safety, and clinical outcomes [15, 45, 46, 47]. Registry data show ECT was given to 2,300 individuals aged >18 years in 2022, increasing in the last decade (Figure 3). Bilateral (BL) ECT constitutes over 80% of treatments [48, 49], although regions vary regarding preferred frequency of treatment and electrode placement [15, 48]. Diagnosis is severe depressive episode in around 75% of cases, while involuntary treatment is allowed in life-threatening clinical situations and constitutes 2% of treatments [15]. A more comprehensive national quality database, including information on clinical response, side effects, and other technical variables, is underway. ECT follows national guidance [50]. Access is free in public hospitals.

### Finland

A 2013 survey had a response rate of 86% [16], giving an ATPR of 231.6 (Table 1). A hitherto unpublished survey covering 2023 saw 8 (32%) of 25 centres respond, covering 39.5% of Finland’s population, reporting on 840 patients who received ECT in 2023 (Supplementary Material). This has been extrapolated to an estimated 2,087 patients nationally and an ATPR of 374.0 (Table 1), tentatively suggesting a steady increase in usage. However, given the low response rate, this has not been included in the comparisons in Figures 2–4. Seventy-seven percent of patients had affective disorders and 13% had schizophrenia, while 46% of patients were outpatients, and 1% were treated without consent. Right unilateral (RUL) placement was most common, followed by BL, typically used when treating schizophrenia or catatonia. Demand is increasing, and maintenance ECT is provided at all centres [51]. Access is free nationwide.

### France

A national registry was implemented in 2017. Use has increased steadily since, particularly in private hospitals, from 2,562 patients in 2017 to 3,972 in 2023 [17]. Compared with psychiatric inpatients who did not receive ECT in 2019, more of the 3,705 patients treated at all 122 centres that year were female, older, socioeconomically advantaged, and experiencing severe or psychotic depressive episodes [52, 53]. There are geographical disparities, only partially explained by distances from ECT centres, with northern and eastern regions tending to treat less frequently [17]. Multicentre studies indicate that ECT is used in accordance with national and international recommendations, but initiation is often delayed: mean episode duration before initiation was 9–10 months, with a mean of 3.4 trials of other treatments [54, 55].

### Germany

An estimate of 62,450 treatments given to 5,700 individuals was extrapolated from 2016 data submitted by 130 (73.0%) of 178 ECT centres that responded to a survey [19], with usage doubling since a previous survey 8 years earlier [18]. Since 2020, the number of treatments has been recorded by the Institute for Hospital Remuneration System (InEK), but there are no more recent data on patient numbers. Nevertheless, 72,193 treatments in 2023 [20] represent an increase of 16% from 62,450 in 2016. Inpatients accounted for 85% of treatments in 2023, while provision varied markedly by federal state [20], demand significantly exceeding capacity in many regions [56]. This increased demand may have been driven in part by a 2012 joint policy statement by national psychiatric associations across German-speaking countries endorsing ECT [57], while the German Association for Psychiatry, Psychotherapy, and Psychosomatics has recently published its own expert consensus statement on ECT for patients lacking capacity [58].

### Italy

There is no national registry and 2024 usage data derive from a survey of eight known centres, conducted for the present study (Supplementary Material). Although the National Bioethics Committee expressed a favourable opinion on ECT in 1995, provided it is administered according to modern standards [59], there are no national clinical guidelines. Instead, a 1999 circular issued by the Ministry of Health provides general recommendations, authorising ECT only for severe, treatment-resistant disorders, exclusively with informed consent [60]. Enduring professional and cultural resistance to ECT means it is reserved for such cases, within a few, primarily northern, centres. Consequently, usage is highly restricted and unevenly distributed. Established practitioners run workshops as part of efforts to reintroduce ECT to more hospitals and turn the tide of steadily diminishing use.

### Netherlands

Health insurance data show 328 patients had ECT in 1999 [23], rising to 1,559 in 2023 (Vektis, personal communication, 5 November 2024), a fourfold increase over 25 years [23] (Figure 3). Clinical practice follows national guidelines [61], which recommend starting ECT with high-dose RUL placement, except when symptoms are severe. The main indication is depressive episode, but it is suggested ECT was underutilised in 2014, when only 1.2% of people with treatment-resistant depression received it [62]. A nationwide platform (Werkgroep ECT Nederland) organises biannual meetings and aims to promote quality and uniformity in practice. ECT training is mandatory for psychiatry residents.

### Norway

Data were sourced from the Norwegian Patient Registry (NPR), complemented by a survey conducted by the National ECT Registry (Supplementary Material), to which 28 of 29 centres responded. These sources showed 1,192 patients had ECT in 2024, when the ECT Registry had just begun patient-level data collection at around half of ECT centres [63]. In 2024, 77% of treatments were administered for unipolar and 15% for bipolar depressive episodes. Mean age was 61 years (range: 18–91), with 63% female. RUL placement was used at 79% of first sessions, with a mean of 8.7 acute treatments. In 187 courses with full Montgomery–Åsberg Depression Rating Scale (MADRS) data, mean scores decreased from 33.1 to 10.3; among 327 courses with post-ECT scores, 51% reached remission (MADRS ≤10) [63]. ECT is exclusively delivered by publicly funded health services.

### Portugal

There are eight private ECT facilities, but most treatments occur in 17 public hospitals. There is no ECT registry; the only available data derive from a hospital registry, which records treatments administered to public hospital inpatients. Data from 2008 to 2015 have been previously reported, with mood disorders accounting for 61% of cases [64]. Prevalence of outpatient and private ECT is unknown, so the degree to which these data underestimate activity is unclear. Consequently, the most recent tally of 204 patients in 2024 (estimated ATPR 19.1) has not been included in the comparison in Figure 2. However, analysis of data from 2016 to 2024 suggests the number of inpatient ECT procedures decreased by 4.4% per year (incidence rate ratio = 0.956, 95% CI: 0.932–0.980, *p* < 0.001) (Figure 3). Access is free at public hospitals.

### Republic of Ireland

A mandatory registry has published annual reports from 2008 to 2024 [29]. Data accuracy is limited by lack of access to registry source material, absence of a national individual health identifier, and the possibility of duplicate data. Annual reports (2008–2023) demonstrate ECT use primarily in affective disorders (mean 79%) and among females (65%), with a mean age of 60 years; 15% of courses involved involuntary treatment, and 78% resulted in Clinical Global Impression–Improvement (CGI–I) outcomes of “much improved” or better. In 2023, 49% of ECT was RUL, 17% was continuation or maintenance, with almost no acute outpatient treatment. Compliance with Rules [65] and a Code of Practice [66] is assessed by annual inspections of centres. All psychiatry trainees must demonstrate competence in ECT administration. The public health system provides free access, but geographical and other inequities persist.

### Spain

Most recent national data are from a survey covering 2012 [31], so they were excluded from the primary comparison. More recent data from Catalonia [67] are likely unrepresentative of the whole country [31].

### Sweden

Usage has remained relatively unchanged in 50 years, with ATPRs around 400 [32]. The Quality Register for ECT is non-mandatory but highly valid, with national coverage of 95% [68]. By cross-linking data with the mandatory National Patient Register for medical procedures, Sweden can present precise information on ECT use. Data encompass details from each course, including patient characteristics and technical parameters, informing research, monitoring of guidelines, and provision of public information concerning practice and outcomes. RUL placement was used in 75%, and depressive episode was the indication in 76% of courses in 2024, with a 46% remission rate (MADRS ≤10) after a median of six treatments, and 76% “much improved” or better on CGI–I [33]. After treatment, 19% had subjective memory impairment, with improvement in 21%. The legal framework for ECT does not differ from other procedures.

### Switzerland

Most recent data derive from a systematic study, identifying 10 centres in 2017, reporting on 402 individuals given 5,192 treatments [34]. ECT has been less prominent in French-speaking cantons [34] but, contrary to previous reports [69, 70], is not legally prohibited and access is improving (Swierkosz-Lenart L, personal communication, 27 March 2025). Recent years have seen marked expansion in provision, with eight ECT centres in 2010 [71, 72] and 2013 [73], increasing to 17 centres in 2024 and 19 in 2025 (Brühl AB, personal communication, 24 September 2025). Practitioners participate in peer support across German-speaking countries.

### United Kingdom

In Scotland, with 8% of the population, all 16 centres mandatorily submitted data on 264 patients to the Scottish ECT Audit Network in 2024 [38]. Activity data in the remaining three regions, comprising 92% of the population, are collected annually by the ECT Accreditation Service, another quality improvement network; in 2024, all 82 centres submitted data, detailing treatment of 2,083 patients. Depressive episode was the indication in 85%, 18% were outpatients, RUL electrode placement was used for 16%, and 52% lacked mental capacity to consent and had ECT under specific legal provisions. Altogether, 2,347 patients were treated at the UK’s 98 centres in 2024. Previous data depict a steady decline over 25 years (Figure 3) [35, 36]. Access is free.

## Discussion

After ECT was first administered in Italy in 1938, its effects were so pronounced that its use had spread across northern and western Europe by 1940 [74]. The modern procedure retains this remarkable efficacy [2, 3] and is safe and well-tolerated when administered correctly [1]. Indeed, modified, brief-pulse ECT is a technologically advanced treatment at the forefront of medical practice in many well-developed healthcare systems.

Despite this, our review, the first collating ECT usage data from Nordic and western European countries, shows marked variation across this high-income region. It brings together findings from various sources to give an up-to-date picture of service provision, complementing a previous review in central and eastern Europe [10].

This review shows three broad patterns of use. First, that seen across the Nordic countries, where the ATPR consistently exceeds 200 patients per million, and ECT is widely considered an early option for depressive episodes and the preferred choice when complicated by psychosis. Second, the pattern observed across the remainder of northwest Europe, from Ireland to Austria, with ATPRs between 30 and 100. In these countries, practice varies between localities, with ECT seen as a last resort by many psychiatrists. Finally, Italy, with an outlying ATPR of 2.7 and huge regional variation in access to ECT, which is only ever proposed in the most dire or life-threatening clinical scenarios.

The reasons for this disparity are complex. Patient attitudes [75, 76] and public perception of ECT, shaped by its profile within fictional and real-world media [77,78], and, in some cases, supposed links to historical government regimes [79], may uniquely affect its use in individual countries or even within different regions of the same country. Public understanding of the true nature of serious mental illness, and its distinction from situational, emotional distress in psychologically vulnerable individuals, is lacking in most societies and leads to scepticism of biological treatments for the former. Appreciation of this distinction among clinicians may vary significantly across the continent, depending upon exposure to training opportunities [80]. There may be a vicious cycle of diminishing access in some locations, whereby a decreasing rate of use, for whatever initial reason, results in closure of centres and loss of training and expertise, perpetuating the downward trend. Lastly, although outside the scope of this article, there are marked differences in legal frameworks that may enable or hinder access to ECT in various countries.

Notably, French-, Dutch-, and German-speaking areas of Europe have seen steadily increasing use in recent years, following espousal by national psychiatric organisations [13, 57, 58, 81, 82, 83, 84], instilling enthusiasm among junior and senior clinicians. Conversely, English-speaking nations have seen a steady decline. Stringent, poorly evidenced UK guidance published in 2003 [85] has been implicated in this decline [36, 86, 87], while there has been a long-running UK-based campaign to discredit ECT [88]. Efforts to reverse this trend include mandatory annual ECT clinic attendance for UK psychiatry residents since 2025, and annual training open to all psychiatrists.

Contrary to previous reports [69, 70, 89, 90], there is no evidence that ECT is prohibited by law in any European country or federal state. Even Slovenia, which has no ECT facilities, permits the treatment in restricted circumstances [91]. It seems likely the word “ban” has been loosely applied by authors to situations where ECT has fallen into disuse or become subject to restrictive guidance [10, 92, 93], then misinterpreted by others as indicative of legal prohibition.

Previous reviews of service provision in the Low Countries have reported the number of individual ECT treatments given per year and calculated population-adjusted usage rates based upon these figures [12, 24, 26, 94]. One [12] inappropriately compared its Belgian rate with an earlier German rate calculated instead using the number of ECT courses [95]. Subsequent researchers have inadvertently misquoted Belgian figures as if they represented the number of patients rather than treatments [7, 10, 27, 32]. The present study sets these comparisons straight.

Rates of RUL placement range from 79% in Norway to just 16% in the United Kingdom, despite high-level evidence confirming RUL as equally effective as BL in depressive episodes, while causing less retrograde amnesia [96]. Depressive episodes were by far the most common indication across the studied region, in contrast with many Asian and eastern European nations, where schizophrenia predominates [7, 10].

Limitations include marked variation in the mode of data collection between countries. Roughly half involved comprehensive registries, with the remainder dependent on questionnaire survey data. Limited response rates to the latter could influence data reliability, while extrapolation assumes non-responding centres have similar activity to responders. Additionally, quantitative data cannot provide detail on clinical decision-making or contextualize how ECT might be under- or over-prescribed in some nations.

The way in which activity is defined differs even amongst countries with comprehensive collection systems. For some, the number of individual patients who received ECT at any time during a calendar year is tallied, while others count only the courses that started or finished in that year. Lastly, data do not distinguish between acute, continuation, and maintenance courses.

## Conclusions

The optimal rate of ECT usage is not known. But widely differing rates across the region suggest this highly effective treatment is underused in some countries with otherwise well-developed healthcare systems, potentially depriving vulnerable patients of vital medical care. Conversely, particularly high usage in some countries underlines the importance of attention to evidence-based indications, ensuring ECT is withheld when other interventions might offer a better risk–benefit balance.

To improve care of seriously ill patients and drive service development, we suggest robust data collection systems are adopted, gathering detailed, accurate, and comprehensive information on ECT practice. Data provision should be straightforward for clinicians, ideally with largely automated transfer of anonymised data from clinic to database. This should improve awareness among clinicians of services and therapeutic outcomes in their localities, reduce stigma, and ultimately lead to higher quality provision.

The international ECT community must come together to support improved patient access in areas with underdeveloped or no services. Along with endorsement of ECT by international psychiatric organisations [97], there may be scope for bodies such as the European Forum for ECT (EFFECT) to collaborate with key stakeholders to ensure appropriate treatment is offered to all patients for whom it is clinically indicated.

## Supporting information

10.1192/j.eurpsy.2026.12238.sm001 Braithwaite et al. supplementary material Braithwaite et al. supplementary material

## Data Availability

Data may be available on request, depending on the relevant country’s legal regulations for the collected data.

## Supplementary material

The supplementary material for this article can be found at http://doi.org/10.1192/j.eurpsy.2026.12238.

## Long descriptions

### Figure 1 Long description

The map highlights 15 European countries in orange with callout boxes providing 3 data points: the number of ECT centres in bold, the data year, and the national population in millions (m).

* In the north:

- Finland: 25 centres, 2023, 5.58m.

- Norway: 29 centres, 2024, 5.57m.

- Sweden: 45 centres, 2024, 10.57m.

- Denmark: 21 centres, 2024, 5.98m.

* In Western and Central Europe:

- United Kingdom: 98 centres, 2024, 69.28m.

- Republic of Ireland: 14 centres, 2024, 5.40m.

- Netherlands: 30 centres, 2023, 17.88m.

- Belgium: 20 centres, 2023, 11.79m.

- Germany: 178 centres, 2016, 82.35m.

- Austria: 12 centres, 2016, 8.74m.

- Switzerland: 10 centres, 2017, 8.45m.

- France: 122 centres, 2019, 65.18m.

* In the South:

- Portugal: 25 centres, 2024, 10.69m.

- Spain: 122 centres, 2012, 46.77m.

- Italy: 8 centres, 2024, 58.99m.

Navigate back to Figure 1.

### Table 1 Long description

The table contains nine columns: Year of data, Reference, Data source, Coverage and details, an empty spacer column, Estimated patients per year, Population in millions, and ATPR (patients per million).

Key data points by country:

* Austria (2016): 271 patients, 8.74m population, 31.0 A T P R.

* Belgium (2023): 1,018 patients, 11.79m population, 86.3 A T P R.

* Denmark (1999 – 2024): Shows a trend of increasing ATPR from 321.4 in 1999 to a peak of 418.6 in 2022, ending at 386.3 in 2024.

* Finland (2023): 2,087 patients, 5.58m population, 374.0 A T P R.

* France (2017 – 2023): ATPR increased from 39.6 to 59.9.

* Germany (2016): 5,700 patients, 82.35m population, 69.2 A T P R.

* Italy (2024): 159 patients, 58.99m population, 2.7 A T P R.

* Netherlands (2023): 1,559 patients, 17.88m population, 87.2 A T P R.

* Norway (2024): 1,192 patients, 5.57m population, 214.0 A T P R.

* Portugal (2024): 204 patients, 10.69m population, 19.1 A T P R.

* Republic of Ireland (2008 – 2024): ATPR decreased from 88.2 to 43.7.

* Spain (2012): 3,090 patients, 46.77m population, 66.1 A T P R.

* Sweden (2010 – 2024): High ATPR ranging from 353.5 to 426.5, ending at 371.4 in 2024.

* Switzerland (2017): 402 patients, 8.45m population, 47.6 A T P R.

* United Kingdom (2024 whole U K): 2,347 patients, 69.28m population, 33.9 A T P R.

Abbreviations include ECT for electroconvulsive therapy and ATPR for annual treated person rate. Data sources vary between surveys, national registers, and insurance data.

Navigate back to Table 1.

### Figure 2 Long description

The vertical Y axis is labelled annual treated person rate (patients per million) with increments of 50 from 0 to 400. The horizontal X axis lists 12 countries with their data year. The data bars from left to right are as follows.

* Austria (2016) 31.0

* Belgium (2023) 86.3

* Denmark (2024) 386.3

* France (2023) 59.9

* Germany (2016) 69.2

* Italy (2024) 2.7

* Netherlands (2023) 87.2

* Norway (2024) 214.0

* Republic of Ireland (2024) 43.7

* Sweden (2024) 371.4

* Switzerland (2017) 47.6

* United Kingdom (2024) 33.9

Denmark and Sweden represent significant peaks in the dataset, while Italy shows the lowest rate.

Navigate back to Figure 2.

### Figure 3 Long description

The X axis represents years from 1999 – 2024. The Y axis represents the annual treated person rate in patients per million, ranging from 0 to 450. The data is divided into two distinct clusters.

High-rate cluster (200 to 450 patients per million):

* Denmark (dark blue solid line): Fluctuates between 260 and 350 until 2017, then rises sharply to a peak of 420 in 2022 before a slight decline.

* Sweden (light green solid line): Starts at 370 in 2010, peaks at 425 in 2015, and remains high between 350 and 400 through 2024.

* Norway (purple solid line): Shows a nearly flat, stable trend around 220 from 2004 – 2024.

Low-rate cluster (0 to 100 patients per million):

* United Kingdom (light green dashed line): Shows a steep decline from 230 in 1999 to approximately 35 by 2024.

* Netherlands (orange solid line): Shows a steady linear increase from 20 in 1999 to nearly 90 in 2023.

* Republic of Ireland (purple dashed line): Fluctuates between 40 and 90 from 2008 to 2024.

* Germany (dark blue dashed line): Shows a gradual increase from 35 in 2008 to 70 in 2016.

* France (light blue solid line): Remains stable at ~ 55 from 2018 – 2023.

* Spain (dark green solid line): Shows a stable trend around 60 from 2000 – 2012.

* Portugal (orange dashed line): Fluctuates at a low level between 20 and 30 from 2015 – 2024.

* Italy (light blue dashed line): Represents the lowest rate, remaining near zero from 2009 – 2024.

Navigate back to Figure 3.

### Figure 4 Long description

A three-panel set of geographical maps.

Panel A, on the left, shows the annual ECT treated person rate per million population using a green color scale. The highest rates, ≥ 300, are in Sweden and Denmark. Norway and Iceland are in the 100 to less than 300 range. The United Kingdom, Ireland, France, and the Netherlands are in the 50 to less than 100 range. Germany and Luxembourg are in the 10 to < 50 range. Italy and Switzerland show the lowest rates at < 10.

Panel B, in the center, shows the compound annual growth rate in ECT use. Red indicates a decrease of less than negative 7 percent, seen in the United Kingdom and Ireland. Orange indicates a decrease of negative 7 percent to less than negative 3 percent, seen in Spain and Italy. Grey indicates a stable rate of negative 3 percent to 3 percent, seen in Norway, Sweden, and France. Light blue indicates growth of 3 percent to < 7 percent, seen in the Netherlands and Belgium. Dark blue indicates growth ≥ 7 percent, seen in Germany.

Panel C, on the right, shows the mean number of patients treated per ECT center per year using a purple color scale. Denmark has the highest density at ≥ 100. Sweden and the Netherlands are in the 75 to < 100 range. Norway, Belgium, and Germany are in the 50 to < 75 range. The United Kingdom, Ireland, France, and Switzerland are in the 25 to less than 50 range. Italy and Iceland have the lowest density at < 25.

Navigate back to Figure 4.
