# Supplementary material for: Use of electroconvulsive therapy in Nordic and western European countries
Source: Eur Psychiatry. 2026 Jul 29;69(1):e84. doi: 10.1192/j.eurpsy.2026.12238 (PMC13735846; doi:10.1192/j.eurpsy.2026.12238)
Supplement: Supplementary file 1 [file S092493382612238Xsup001.docx]

**Survey methodologies**

**Austria**

This cross-sectional study, conducted in 2017, formed part of an interdisciplinary and international collaboration between the Department of Psychiatry, Psychotherapy and Psychosomatics at RWTH Aachen University Hospital (Germany), the Institute of History, Theory and Ethics in Medicine at RWTH Aachen University Hospital (Germany), the Department of Adult Psychiatry at the Regional Hospital of Rankweil (Austria) and the Department of Psychiatry at San Maurizio Hospital in Bolzano (Italy). A standardised questionnaire was used to survey the prevalence and practice of ECT in Austria. The same questionnaire was previously used for surveys in Germany [19], Italy [22] and Switzerland [34]. A 15-item German-language digital fillable document was sent to public and private ECT centres.

ECT centres were selected based upon those already identified by previous publications in the years 2000 [39], 2003-2004 [40] and 2013 [41], as well as current notifications from the German Association for Psychiatry, Psychotherapy and Psychosomatics regarding medical facilities offering ECT in German-speaking countries, and personal communication. In the second half of 2017, 44 healthcare facilities in Austria were first contacted by email, followed up by a telephone call. Of these facilities, 23 (52%) responded to the questionnaire, 12 of which used ECT. These 12 facilities formed the basis of the sample for the present study. None of the 21 facilities that did not respond to the survey were known, via the above sources, to be providers of ECT. Consequently, the 12 ECT centres that did respond most likely represented 100% of existing centres nationally. For the purposes of the survey, respondents were not asked to access any individual patient data. Rather, they were asked, amongst other questions, how many patients were treated at their centre per year. Centres most likely provided the relevant total for 2016 (the most recent complete calendar year at the time of the survey), but some may have used an average of aggregated data from more than one recent year. The data thus provided were not verified.

**Belgium**

In an ongoing, hitherto unpublished survey, the practice of ECT in Belgium is being studied. Belgium is a federal state, divided into three regions: Flanders, the Brussels Capital Region, and Wallonia. There is no specified accreditation system for ECT. Using data from the National Institute for Health and Disability Insurance, all 20 hospitals that invoiced for at least one ECT session in 2023 were identified. A questionnaire, adapted from the one used in a previous survey [13], was sent to the responsible psychiatrist at each ECT centre in April 2025. After a few weeks, these psychiatrists were contacted by telephone, to again request their participation. Respondents were not asked to access any individual patient data for the purpose of this study. Instead, they were asked to submit data that had already been collated within their centre for service evaluation purposes.

Each of the six largest ECT centres responded, providing both the number of individual ECT treatments and the number of unique patients treated in 2023. Together, these six centres accounted for 11,705 (77.8%) of the 15,046 ECT treatments administered nationally (**eFigure 5**), as identified by the insurance data. The mean number of treatments per patient across these six centres was calculated (total 11,705 treatments; total 792 patients; mean 14.78 treatments per patient). The total number of treatments recorded in the insurance data (15,046) was divided by this mean number of treatments per patient (14.78), to estimate the number of unique patients treated nationally (1,018).

**Finland**

The survey was carried out by authors SK and KJ, with Lauri Virtanen (Turku University Hospital), Anu Holm (University of Turku), Niko Seppälä (Satakunta Wellbeing Service County, Pori), Petri Sumia (Tampere University Hospital) and Erika Jääskeläinen (University of Oulu). Registry study Permit (121/2023) was granted from the Wellbeing Services County of Northern Ostrobothnia for the survey. A structured questionnaire was created by SK, LV, AH, KJ, NS and PS, with help from Marjut Grainger (Finnish Institute for Health and Welfare) using the Webropol online platform (<https://webropol.co.uk/>), and was used for collecting the data. It included a total of 171 questions, both in the form of open questions and in slider-scales and multiple-choice answers, about electroconvulsive therapy (ECT), repetitive transcranial magnetic stimulation (rTMS), transcranial direct current stimulation (tDCS), and intravenous and intranasal esketamine. The questions were arranged by treatment modality and if a specific treatment was not given in the unit, the following questions about that treatment were skipped. For each treatment modality, questions included treatment numbers, patient numbers, patients’ diagnoses, age distribution, and treatment parameters. Respondents were not asked to access any individual patient data for the purpose of this study. Instead, they were asked to submit data that had already been collated within their clinic for service evaluation purposes.

The questionnaire was sent in April 2024 to a Finnish ECT-email-post list, which includes the email addresses of most of the neuromodulation units and other units offering ECT, rTMS, tDCS, iv-ketamine, and in-esketamine. To increase the number of answers, the questionnaire was emailed again several times to the posting list, during April 2024 – November 2025. In addition, the questionnaire was emailed to the chief physicians in wellbeing counties, if answers were missing in certain counties/areas, and it was advertised during national training workshops and lectures on ECT. Further, to also receive answers from smaller private units, the questionnaire was sent to private medical centres in October – November 2024. We received a total of 12 answers. Data from the eight centres providing ECT were used for the present study. The numerical data were analysed with the tools in Webropol and Microsoft Excel, to count means, medians and standard deviations. Open answers were grouped into categories as appropriate.

**Italy**

The survey was devised and conducted by CIC. A standardised questionnaire was used to gather data on national ECT usage. The list of survey recipients was procured through a combination of direct knowledge of clinical leads and references from previous peer-reviewed work that identified active ECT centres in earlier years [22]. Although some centres identified in older literature are no longer active, this approach ensured a comprehensive identification of the current national landscape. The survey was conducted during the second half of 2025. Clinics were contacted via email and telephone. Respondents were not asked to access any individual patient data for the purpose of this survey. Instead, they were asked to submit aggregated data that had already been collated within their centre for service evaluation purposes. Follow-up prompts were issued in September 2025 and again in February 2026.

A total of eight active ECT centres were identified. Of these, seven (Brunico, Pisa, Rome, Bologna, Brescia, Milan, and Oristano) provided data, resulting in a centre response rate of 87.5%. However, the clinic in Verona, which did not provide a response to the present survey, was by far the single largest ECT centre in an earlier survey, accounting for 110 (37.54%) of 293 patients in 2017 [22]. Consequently, the total number of patients from the seven responding centres in the current survey (99 patients) was extrapolated using a divisor of 0.6246, to estimate a total of 159 patients. No further data manipulation or transformation was undertaken.

**Norway**

The annual national survey of ECT service was conducted by the management team of the National ECT Registry at Haukeland University Hospital. The registry administration was responsible for distributing the survey, monitoring responses, and ensuring data quality. The list of survey recipients was compiled using the registry’s continuously updated overview of all active ECT‑providing units in Norway. All ECT‑providing centres were contacted by email in spring 2025 and given follow-up prompts thereafter. Centres were asked to provide aggregated service-level information for 2024 on the number of patients treated with ECT, the number of treatment sessions delivered, technical and organisational characteristics of ECT provision, staffing, equipment, and adherence to national guidelines.

Further, we asked all treatment centres to describe their coding practice to the Norwegian Patient Register (NPR). We used the NPR as the primary source to estimate the number of patients receiving ECT in Norway in 2024. However, for units that self‑reported gaps in coding to NPR, or when NPR data conflicted with contextual information (e.g., declared treatment capacity), we triangulated NPR data with unit self‑reports and, where warranted, replaced NPR counts with survey‑reported counts. Consequently, the number of treated patients is a best‑estimate synthesis from multiple sources. Respondents were not asked to access or extract any form of individual patient data for the purposes of the survey. The response rate was 28 (97%) of 29 centres.

**Methodologies of peer-reviewed publications**

**Germany**

Data for the primary analysis are derived from a survey, the methodology of which is fully described in a peer-reviewed publication [19].

**Spain**

The most recent data available were not used in the primary analysis due to their age, but the methodology of the survey from which those earlier data are derived is fully described in a peer-reviewed publication [31].

**Switzerland**

Data for the primary analysis are derived from a survey, the methodology of which is fully described in a peer-reviewed publication [34].

**Methodologies of non-survey administrative datasets**

**Denmark**

Healthcare interventions are recorded by healthcare providers onto the National Patient Register (Landspatientregisteret). Individual ECT treatments are therefore immediately logged alongside a unique patient identifier. Anonymised data from the register are held by Statistics Denmark (Danmarks Statistik, DS), which was contacted for the purpose of the present study, and on 5 March 2026 supplied the numbers of patients who received ECT in each year from 2003 to 2023. DS requested that, for publication, values be rounded to the nearest 10. Consequently, values for the years 2008 to 2014 were instead taken from a peer-reviewed publication using the same data source that had been permitted to use unrounded values for those years [15].

**France**

A national administrative database, Programme de Médicalisation des Systèmes d’Information (PMSI), is managed by the Technical Agency for Hospital Information (Agence Technique de l'Information sur l'Hospitalisation) and collects data on hospital activity including specific procedures. ECT usage data for the present study are derived from a peer-reviewed publication that accessed the PMSI database, which includes further information on the methodology of data procurement [17].

**Netherlands**

Vektis is the national healthcare information and business intelligence centre for the healthcare system. It includes detailed data on all healthcare delivered to patients with healthcare insurance, which is universal and mandatory for all residents. Although established to enable reimbursement of healthcare providers by insurers, it also provides anonymised health data for the purpose of research. It was contacted for the purpose of the present study and on 5 November 2024 supplied the number of patients who received ECT in 2023. Data on ECT activity covers patients treated under the Healthcare Insurance Act (Zorgverzekeraarswet), but not patients treated under the Long-term Care Act (Wet langdurige zorg) or those in forensic care. This results in a slight underestimation of ECT activity.

**Portugal**

An administrative database is provided by the Health System Central Administration (Administração Central do Sistema de Saúde, ACSS), containing data on all public hospital inpatient admissions. This is designed to calculate reimbursement costs for the 70% of admissions that take place within the public sector [64]. International Classification of Diseases (ICD) codes are used by doctors to specify procedures, such as ECT.

**Republic of Ireland**

All ECT centres submit anonymised clinical data to a mandatory registry administered by the Mental Health Commission (MHC). This is done using a secure online form following completion of a course of ECT. Further information on the methodology of MHC data collection is available in the annual MHC data reports: <https://www.mhcirl.ie/publications/>.

**Sweden**

All ECT centres submit anonymised clinical data to the Quality Register for ECT (Kvalitetsregister ECT, Q-ECT). Some centres use a secure online form following completion of a course of ECT, while other centres have automatic linking of patient records to the Q-ECT. Further information on the methodology of Q-ECT data collection is available in the annual Q-ECT data reports: <https://ect.rcnorr.se/rapporter/arsrapporter/>.

**United Kingdom**

In England, Scotland and Wales (92% of the population), all ECT centres hold membership of the ECT Accreditation Service (ECTAS). In 2024, all ECT centres submitted anonymised clinical data to ECTAS, using a secure online form following completion of each ECT course. Further information on the methodology is available in the annual ECTAS data reports: <https://www.rcpsych.ac.uk/improving-care/ccqi/quality-networks-accreditation/ectas/publications-and-links>. In Scotland (8% of the UK population), data submission to the Scottish ECT Audit Network (SEAN) is mandatory. In 2024, all ECT centres submitted anonymised clinical data to SEAN by via a secure online form following completion of each individual ECT treatment. Further information on the methodology is available in the annual SEAN data reports: <https://publichealthscotland.scot/publications/show-all-releases?id=49325>.

**Copyright notice**

All maps are adapted from: <https://commons.wikimedia.org/wiki/File:Blank_political_map_Europe_in_2006_WF.svg>, which is in the public domain.

**Supplementary references**

e98.	Asztalos M, Ungvari GS, Gazdag G. Changes in Electroconvulsive Therapy Practice in the Last 12 Years in Hungary. J ECT. 2017 Dec;33(4):260-263. <https://doi.org/10.1097/YCT.0000000000000428>

e99.	Antosik-Wójcińska AZ, Dominiak M, Mierzejewski P, Jażdżyk P, Gazdag G, Takacs R, Wichniak A. Changes in the Practice of Electroconvulsive Therapy in Poland: A Nationwide Survey Comparing Data Between 2005 and 2020. Neuropsychiatr Dis Treat. 2021 Feb 22;17:605-612. <https://doi.org/10.2147/NDT.S296210>

**eFigure 5.** Number of ECT treatments ("sessions") given in Belgium across all 20 ECT centres in 2023.

Bxl, Brussels Capital Region; N, number of ECT centres.

**eFigure 6.** Compound annual growth rate in electroconvulsive therapy use.

Values calculated using different time periods, as indicated, due to varying data availability. Belgium value based on numbers of treatments, not numbers of patients. Exclusions: Austria and Switzerland (data only available for one timepoint); Finland (low response rate for most recent survey).

**eTable 2.** Annual treated person rates (patients per million) between the years 1999 and 2024.

|  | **1999** | **2000** | **2001** | **2002** | **2003** | **2004** | **2005** | **2006** | **2007** | **2008** | **2009** | **2010** | **2011** | **2012** | **2013** | **2014** | **2015** | **2016** | **2017** | **2018** | **2019** | **2020** | **2021** | **2022** | **2023** | **2024** |
| --- | --- | --- | --- | --- | --- | --- | --- | --- | --- | --- | --- | --- | --- | --- | --- | --- | --- | --- | --- | --- | --- | --- | --- | --- | --- | --- |
| **Austria** |  |  |  |  |  |  |  |  |  |  |  |  |  |  |  |  |  |  | 31.0 |  |  |  |  |  |  |  |
| **Belgium** |  |  |  |  |  |  |  |  |  |  |  |  |  |  |  |  |  |  |  |  |  |  |  |  | 86.3 |  |
| **Denmark** | 321.4 |  |  |  | 261.6 | 288.9 | 330.3 | 327.2 | 326.0 | 306.9 | 323.9 | 344.9 | 326.8 | 296.4 | 303.9 | 326.1 | 316.9 | 338.6 | 328.1 | 366.5 | 402.8 | 396.2 | 406.1 | 418.6 | 406.7 | 386.3 |
| **Finland** |  |  |  |  |  |  |  |  |  |  |  |  |  |  | 231.6 |  |  |  |  |  |  |  |  |  |  |  |
| **France** |  |  |  |  |  |  |  |  |  |  |  |  |  |  |  |  |  |  | 39.6 | 55.0 | 57.1 | 48.9 | 53.7 | 53.7 | 59.9 |  |
| **Germany** |  |  |  |  |  |  |  |  |  | 34.1 |  |  |  |  |  |  |  | 69.2 |  |  |  |  |  |  |  |  |
| **Italy** |  |  |  |  |  |  |  |  |  |  | 5.9 |  |  |  |  |  |  |  | 4.9 |  |  |  |  |  |  | 2.7 |
| **Netherlands** | 20.7 |  |  |  |  |  |  |  |  |  |  |  |  |  |  | 57.3 | 57.0 |  |  |  |  |  |  |  | 87.2 |  |
| **Norway** |  |  |  |  |  | 219.6 |  |  |  |  |  |  |  |  |  |  |  |  |  |  |  |  |  |  |  | 214.0 |
| **Portugal** |  |  |  |  |  |  |  |  |  |  |  |  |  |  |  |  |  | 32.4 | 25.2 | 23.7 | 25.8 | 20.1 | 23.8 | 23.7 | 21.0 | 19.1 |
| **Republic of Ireland** |  |  |  |  |  |  |  |  |  | 88.2 | 79.7 | 73.2 | 69.0 | 53.0 | 55.6 | 59.7 | 51.7 | 55.3 | 53.2 | 57.6 | 57.4 | 47.4 | 42.9 | 39.5 | 38.6 | 43.7 |
| **Spain** |  | 60.0 |  |  |  |  |  |  |  |  |  |  |  | 66.1 |  |  |  |  |  |  |  |  |  |  |  |  |
| **Sweden** |  |  |  |  |  |  |  |  |  |  |  | 367.0 |  | 404.2 | 413.5 | 421.5 | 426.5 | 399.4 | 395.3 | 407.3 | 388.5 | 353.5 | 368.3 | 378.2 | 380.1 | 371.4 |
| **Switzerland** |  |  |  |  |  |  |  |  |  |  |  |  |  |  |  |  |  |  | 47.6 |  |  |  |  |  |  |  |
| **United Kingdom** | 231.3 |  |  | 182.9 |  |  |  | 100.1 |  |  |  |  |  | 38.8 |  | 38.8 |  |  |  |  |  |  |  | 33.9 |  | 33.9 |

**eTable 3.** Number of ECT centres and estimated annual treated person rates (patients per million) in European countries.

|  | **Country** | **Reference** | **Year(s) of data** | **Population (m)** | **Number of ECT centres** | **ATPR (patients per million)** |
| --- | --- | --- | --- | --- | --- | --- |
|  |  |  |  |  |  |  |
|  | Austria | Unpublished | 2016 | 8.7 | 12 | 31 |
|  | Belgium | Unpublished | 2023 | 11.8 | 20 | 86 |
|  | Bulgaria | Gazdag et al. (2017) [10] | 2010 | 7.3 | 4 | 16 |
|  | Croatia | Gazdag et al. (2017) [10] | 2012-2013 | 4.3 | 3 | 13 |
|  | Czechia | Gazdag et al. (2017) [10] | 2010-2011 | 10.5 | 28 | 136 |
|  | Denmark | DNPR, pc, 5 Mar 2026 | 2024 | 6.0 | 21 | 386 |
|  | Estonia | Gazdag et al. (2017) [10] | 2010 | 1.3 | 5 | 278 |
|  | Finland | Sumia et al. (2021) [16] | 2013 | 5.4 | 29 | 232 |
|  | France | Barruel et al. (2025) [17] | 2023 | 66.3 | ^a^ 122 | 60 |
|  | Germany | Jolivet & Grözinger (2021) [19] | 2016 | 82.4 | 178 | 69 |
|  | Hungary | Asztalos et al. (2017) [e98] | 2014 | 9.8 | 22 | 18 |
|  | Italy | Unpublished | 2024 | 59.0 | 8 | 3 |
|  | Latvia | Gazdag et al. (2017) [10] | 2010 | 2.0 | 2 | 5 |
|  | Lithuania | Gazdag et al. (2017) [10] | 2010 | 3.2 | 5 | 38 |
|  | Netherlands | Vektis, pc, 5 Nov 2024 | 2023 | 17.9 | 30 | 87 |
|  | Norway | NPR, pc, 10 Dec 2025; unpublished | 2024 | 5.6 | 29 | 214 |
|  | Poland | Antosik-Wójcińska et al. (2021) [e99] | 2019 | 38.0 | 19 | 13 |
|  | Republic of Ireland | MHC (2025) [29] | 2024 | 5.4 | 14 | 44 |
|  | Serbia | Gazdag et al. (2017) [10] | 2012 | 7.2 | 1 | 8 |
|  | Slovakia | Gazdag et al. (2017) [10] | 2008-2010 | 5.4 | 33 | 292 |
|  | Slovenia | Gazdag et al. (2017) [10] | N/A | 2.1 | 0 | 0 |
|  | Spain | Vera et al. (2016) [31] | 2012 | 46.8 | 122 | 66 |
|  | Sweden | Q-ECT (2025) [33] | 2024 | 10.6 | 45 | 371 |
|  | Switzerland | Wilhelmy et al. (2023) [34] | 2017 | 8.5 | 10 | 48 |
|  | Ukraine | Gazdag et al. (2017) [10] | 2011 | 45.5 | 8 | 2 |
|  | United Kingdom | SEAN (2025) [38]; ECTAS, pc, 5 Mar 2026 | 2024 | 69.3 | 98 | 34 |
|  |  |  |  |  |  |  |

Values rounded in keeping with values presented in Gazdag et al. (2017) [10]. Newer data may exist for some central and eastern European countries. Portugal excluded due to restriction of scope of data to public hospital inpatients. ECT, electroconvulsive therapy; ATPR, annual treated person rate; pc, personal communication; DNPR, Danish National Patient Register; NPR, Norwegian Patient Registry; MHC, Mental Health Commission; Q-ECT, Kvalitetsregister ECT; SEAN, Scottish Electroconvulsive Therapy Audit Network; ECTAS, Electroconvulsive Therapy Accreditation Service.

^a^ Number of clinics in France from year 2019 [52]; number from year 2023 not available in primary source [17].

**eFigure 7.** Estimated annual treated person rates (patients per million) in European countries.

Dark blue bars represent data meeting inclusion criteria for the present study i.e. from Nordic and western European countries from after 1 Jan 2015. Light blue bars represent other data. Year(s) of data in brackets; Slovenia has no year of data due to total lack of activity. Sources of data as specified in eTable 3. Newer data may exist for some central and eastern European countries. Portugal excluded due to restriction of scope of data to public hospital inpatients.
